# Sex, personality and conspecific density influence natal dispersal with lifetime fitness consequences in urban and rural burrowing owls

**DOI:** 10.1371/journal.pone.0226089

**Published:** 2020-02-12

**Authors:** Álvaro Luna, Antonio Palma, Ana Sanz-Aguilar, José L. Tella, Martina Carrete

**Affiliations:** 1 Department of Conservation Biology, Estación Biológica de Doñana—CSIC, Sevilla, Spain; 2 Animal Demography and Ecology Unit, IMEDEA (CSIC-UIB), Esporles, Spain; 3 Applied Zoology and Conservation Group, University of Balearic Islands, Palma, Spain; 4 Department of Physical, Chemical and Natural Systems, Universidad Pablo de Olavide, Sevilla, Spain; Universitat Autònoma de Barcelona, SPAIN

## Abstract

There is a growing need to understand how species respond to habitat changes and the potential key role played by natal dispersal in population dynamics, structure and gene flow. However, few studies have explored differences in this process between conspecifics living in natural habitats and those inhabiting landscapes highly transformed by humans, such as cities. Here, we investigate how individual traits and social characteristics can influence the natal dispersal decisions of burrowing owls (*Athene cunicularia)* living in urban and rural areas, as well as the consequences in terms of reproductive success and apparent survival. We found short dispersal movements among individuals, with differences between urban and rural birds (i.e., the former covering shorter distances than the latter), maybe because of the higher conspecific density of urban compared to rural areas. Moreover, we found that urban and rural females as well as bold individuals (i.e., individuals with shorter flight initiation distance) exhibited longer dispersal distances than their counterparts. These dispersal decisions have effects on individual fitness. Individuals traveling longer distances increased their reproductive prospects (productivity during the first breeding attempt, and long term productivity). However, the apparent survival of females decreased when they dispersed farther from their natal territory. Although further research is needed to properly understand the ecological and evolutionary consequences of dispersal patterns in transformed habitats, our results provide information about the drivers and the consequences of the restricted natal movements of this species, which may explain its population structuring through restricted gene flow between and within urban and rural areas.

## Introduction

Natal dispersal, defined as the movement of individuals from their birthplace to their first breeding area [1] may influence the future survival, fecundity, and lifetime fitness of individuals [2,3,4], playing an important role in the evolution, persistence and spread of populations and species[5,6,7,8,9,10,11]. Thus, a large number of studies have investigated the factors driving natal dispersal decisions, in particular the importance of social and environmental cues (e.g. conspecific density and habitat characteristics; [12,13], previous experience [14]) and phenotypic attributes, including personality [15,16], structural size [17,18], body mass [13,19] and sex [20], among others (reviewed by [21]). All of these factors can interact in complex and subtle ways, varying across the spatial range of a species, such that natal dispersal decisions, as well as their conditioning, can differ among species but also among populations of the same species [21]. Therefore, studies comparing the dispersal patterns of conspecifics inhabiting areas with contrasting characteristics and subjected to different selection regimes are important to better understand the dynamic nature of dispersal as well as how drivers of global change affect the fate of animal populations.

Urbanization is the most drastic and persistent alteration of a landscape, creating new habitats starkly different from the natural habitats it replaces [22]. Currently, the urban expansion is occurring at an unprecedented rate, mainly by the migration from rural to urban areas. One century ago, only 10% of humans inhabited cities, and today about the 50% did so, with a 70% predicted to live in urbanized landscapes by 2050 [22, 23]. Furthermore, the continuous human population growth (from today’s 7 billion people to the estimated 9 billion by 2050 [24]) also contribute to the large increase in the spatial extent of urbanized areas worldwide. Although urbanization leads to an overall loss of biodiversity (the so-called ‘biotic homogenization process; [25, 26, 27]), some species seem to prosper in these environments [28]. Among birds, for example, nearly 20% of the roughly 10,000 described species can be found in cities [29]. Thus, understanding the factors that allow them to persist within these landscapes as well as the consequences for their population dynamics and structure is as important as identifying the drivers of species loss. A common finding of studies exploring traits that allow individuals to live in urban environments is that urban birds are less fearful of humans (bolder) than their rural counterparts, such that urban colonization may be driven by tame individuals from species with high interindividual variability in fear of humans crossing the disturbance frontier [30,31,32,33,34,35]. Fear of humans, measured as the distance at which an individual flees in response to an approaching person (also called flight initiation distance, hereafter FID), is repeatable throughout the adult lifespan [36, 37], heritable [38], and correlates with other behaviors such as exploration and antipredatory response [34]. Thus, it can be considered a personality trait [39]. Another common pattern found in the literature is that urbanization leads to a reduction in predators [40, 41], such that species able to colonize urbanized habitats can show larger densities or abundances than their rural counterparts [42,43,44,45]. These changes in individual behaviors or personalities, conspecific density and predation pressure can have profound effects on the breeding performance and survival prospects of individuals [33], including their dispersal decisions [39]. There is a growing literature showing how the dispersal patterns of some species change in response to local conditions and depending on the phenotypic characteristics of the individuals present in a particular population [6, 21]. Despite this, there are no studies exploring whether urban individuals show different natal dispersal movements than their rural counterparts.

Here, we use the burrowing owl (*Athene cunicularia)* as a study model to assess the role played by individual characteristics (i.e., sex, and personality), and the environment where an individual was born (i.e., the breeding density and productivity of conspecifics) on the natal dispersal distances of urban and rural individuals. We predict that if natal dispersal is related to individual personality, bold and shy individuals (i.e., those with short and large FID, respectively) will breed at different distances from the sites where they were born. Some studies show that boldness is positively associated with dispersal tendency [15, 46, 47, 48] and thus, urban birds (which are bolder than rural individuals; [32, 33, 34,35]) should have longer natal dispersal distances than rural ones. However, our previous work has indicated that the breeding dispersal propensity of burrowing owls is personality-dependent among rural but not urban individuals, with shy birds moving further than bold ones but only in the rural habitat [39]. Moreover, as avian females usually move greater distances during dispersal than males [49, 50], we expect to find this general pattern among all urban and rural individuals. Social features such as conspecific density and productivity can be used by individuals as indicators of habitat quality, such that dispersers would prefer to move to high-density and high-productivity areas [51, 52, 53]. However, young individuals born in very dense areas could also disperse to low-density areas to avoid intraspecific competition [54, 55]. In our study model, predation, the main determinant of individual fitness [33, 41], is highly unpredictable; thus, conspecific presence and productivity can be used as indicative clues of predation risk. We expect that individuals born in areas with low conspecific density and/or productivity move to other areas of higher quality (i.e., high conspecific density and/or productivity). As urban areas have fewer predators than rural ones [41], this pattern is expected to be more pronounced among birds living in more natural areas. Finally, we evaluated posterior survival and reproductive output. We predict that due to the risk associated with moving to areas far from their natal sites, where individuals are not familiar with the habitat and predation is difficult to assess, birds moving greater distances should have lower reproductive output and survival than those staying close to their natal areas [4].

## Material and methods

### Study system and field procedures

The study area covers approximately 5,400 km^2^ of natural grasslands, pastures and cereal crops, surrounding the city of Bahía Blanca, Argentina [30, 36]. We surveyed burrowing owls from 2006 to 2018, monitoring ca. 2500 and ca. 3200 urban and rural nests, respectively. Urban nests were located in private and public gardens, vacant lots among houses, curbs of the streets, roundabouts, and large avenues, in contact with the intense daily activity derived from cities. Rural nests, on the contrary, were located in large extensions of natural or semi-natural grasslands, with very low human presence. It is worth noting that the city is immediately surrounded by large areas of pastures, and there is no obstacle precluding the movement of individuals between urban and rural areas. Moreover, as these owls are able to excavate their own burrows, their distribution is not constrained by the availability of nesting structures.

During the breeding period (from November to early February), all known breeding sites and areas of suitable habitat for the species were regularly visited, and active nests were georeferenced using a portable GPS. Chicks were captured at their natal nests using bow nets and ribbon carpets, and marked with plastic colored and numbered rings readable at a distance. Resightings of marked birds were done annually during the breeding period, throughout the study area [33, 36]-. At the end of every breeding season (except in 2018), we recorded the productivity of each nest as the number of young that reached fledging age.

Natal dispersal distance was measured as the straight line between an individual’s nest site and its first breeding nest. Individuals that were not observed in their first but in their second breeding season were included in our analyses, using as natal dispersal distance the straight line between their natal site and their second breeding nest. In these cases, we assumed that natal dispersal distances were larger than the short breeding dispersal distances recorded for the species [39], such that the breeding location at the second nest would not be far from the first breeding point. However, we cannot discard the possibility that those birds were actually breeding for their first time at 2 years of age, and that this dispersal distance corresponds to their natal dispersal.

We sexed adult birds based on plumage pattern and coloration [36] and, when needed, by molecular procedures [44]. FID was measured using the standard procedure of walking toward undisturbed individuals perched close to their nests during the day and recording the distance at which the bird flew away [38]. We used one FID per individual or the mean when more than one value was available, given the high repeatability of this behavior (r = 0.91; [35]). Conspecific density was calculated using an annual aggregation index for each breeding pair, obtained as their relative position within the spatial distribution of all breeding pairs [56]. This index reflects conspecific density and was obtained using the GPS location of all breeding pairs as *Si* = *Σ exp* (−*dij*) (with *i* ≠ *j*), where *dij* was the linear distance between pairs *i* and *j*. We also estimated the productivity of the breeding pairs in the surroundings of each breeding site using a modification of this aggregation index, where the distance of each breeding pair was weighted by its productivity. Conspecific productivity was then obtained as the residual of this last variable against the aggregation index calculated previously.

### Ethics statements

Fieldwork and procedures were conducted under permits from the Argentinean wildlife agency (22500-4102/09), and the owners of private properties, in accordance with the approved guidelines of the Ethics Committee of CSIC (CEBA-EBD-11-28).

### Statistical approach

We used Generalized Linear Mixed Models (GLMM) to assess the effects of individual traits (sex and personality, measured as FID), and social variables (conspecific density and productivity in the natal area) on the natal dispersal distances (log-transformed, identity link function, normal error distribution) of urban and rural burrowing owls. Due to the low proportion of birds that bred for the first time in their natal nests (philopatric individuals), dispersal distance was modeled as a continuous variable ranging from 0 to the maximum distance observed. Models were built using a different combination of variables in interaction with habitat (urban or rural) but including alternatively only one descriptor of the social environment (conspecific density or productivity) due to their slight, but significant, correlation (Spearman correlation: rho = 0.41, p<0.0001). All models included the year as a random variable. Although individuals born in the same clutches share the same social environment and habitat and can even show similar FID [38], clutch identity was not considered as a random term in models because only 20% of individuals belong to shared clutches (45 individuals of 22 clutches). Thus, the dimension of the variance-covariance matrix was exactly zero and model comparissons using likelihood ratio (LRT) tests did not support the inclusion of this random term in models.

We then compared the social environment of individuals (conspecific density and productivity; identity link functions, normal error distributions) between natal and dispersal sites. Due to differences in conspecific density and productivity between rural and urban areas, we included the habitat of recruitment as a factor in the models. We tested whether these differences were related to the individuals' dispersal distances, again considering potential habitat differences. All models included the year as a random variable to control for interannual differences.

Next, we assessed the effect of dispersal distance on immediate (the first breeding event) and long term productivity of individuals (log link functions, negative binomial error distributions). For long term productivity analyses we only considered individuals with known reproductive output for every year during their reproductive careers and not seen during at least 2 years prior to the end of this study (until 2016), which had a very high probability of being dead (probability of not resighting an alive individual over 2 years at least once was 0.005 for males and 0.033 for females). Models included the dispersal distance of individuals, the habitat where the bird bred, and its sex. Models obtained for long term productivity also included, for each year (random term), the age of each bird (covariate) and its identity (random term) to control for potential improvements along years and pseudoreplication [4]. We also considered potential habitat and sexual differences by including the interaction between sex and dispersal distance and between habitat and dispersal distance in models.

Model selection was performed using the Akaike Information Criterion corrected for small sample sizes (AICc; [57]). Models within 2 AICc units of the best one were considered as alternatives and used to perform model averaging (MuMIn package, [58]). All continuous variables were centered and standardized before modeling to properly estimate their main effects regardless of whether we included the interaction [59]. We considered that a given effect received no, weak or strong statistical support when the 95% confidence interval (CI) strongly overlapped with zero, barely overlapped with zero, or did not overlap with zero, respectively. Finally, we calculated the coefficient of determination, R^2^, as a measure of the variance explained by a model [60]. Statistical analyses were conducted in R 3.1.2 ([61] using the package “lme4” [62]).

We modeled apparent survival following basic capture–mark–recapture methods for open populations, in which return rates were corrected for detection (p) probabilities [63]. For this purpose, we created encounter histories for every marked individual with known natal dispersal distances. We evaluated if adult apparent survival was related to natal dispersal distance (log transformed) by including it as an individual covariate while habitat (rural and urban) and sex were considered as factors. We first tested the effects of time, habitat and sex on detection probability and, once the best structure for this parameter was selected, we modeled survival. Models differing by < 2 AICc points were considered equivalent [57]. We tested the goodness-of-fit of our global model using the program U-CARE [64]. Models were implemented in the program E-SURGE 2.1.4 [65].

## Results

**Fig 1:**
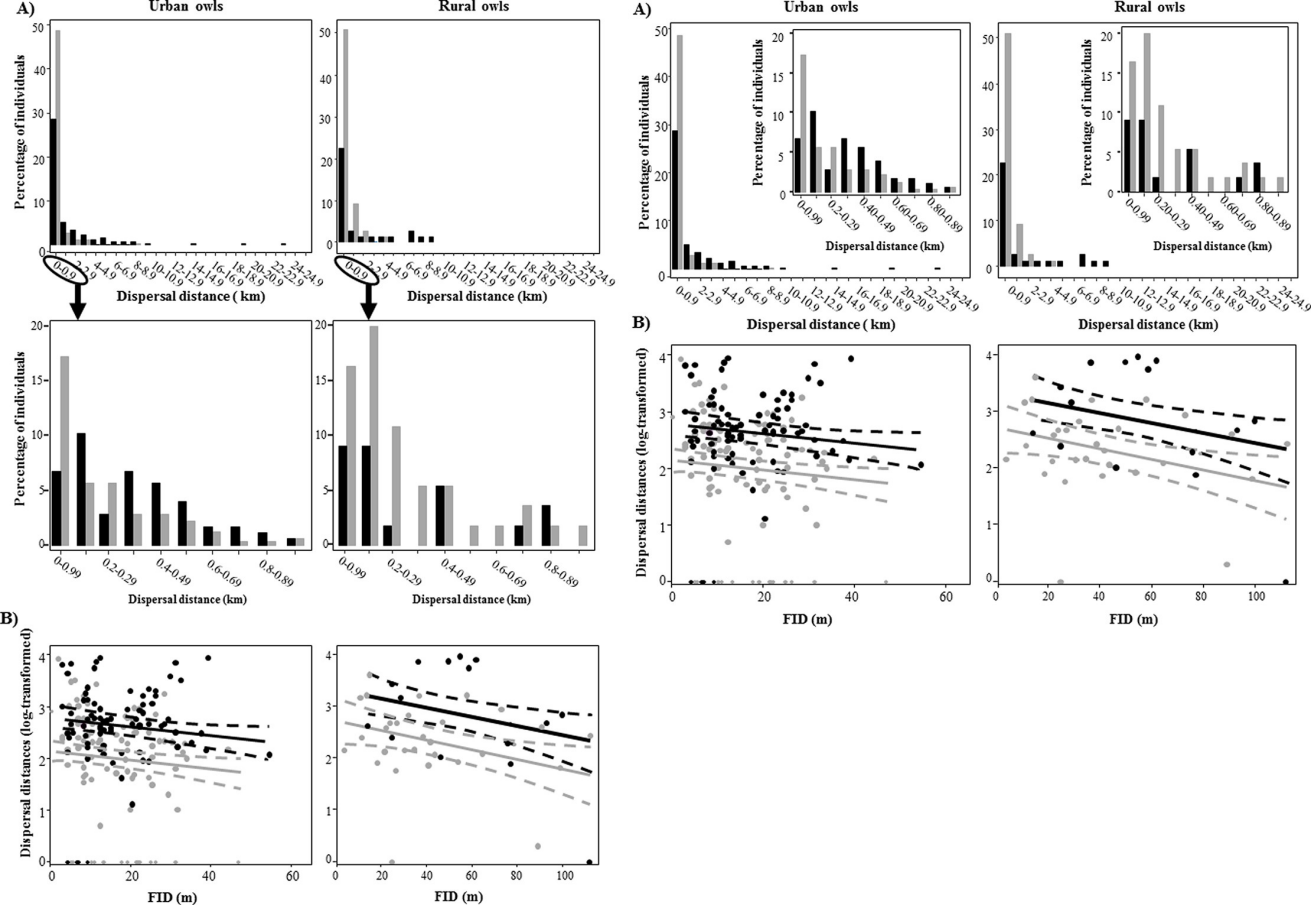
A) Natal dispersal distances of urban and rural burrowing owls *Athene cunicularia* (males: gray bars; females: black bars). The inserted figure shows a detailed distribution of dispersal distances lower than 1km. B) Relationship between natal dispersal distances (log-transformed) and individual personality (measured as FID, flight initiation distance). Lines show the tendency observed for males (gray line) and females (black line). Dashed lines represent the 95% confidence interval. Dots are raw data (males: gray dots, females: black dots).

During the study period, we marked 830 urban and 566 rural burrowing owls with PVC rings, and were able to record 321 natal dispersal events in 75 rural (48 males and 27 females) and 246 urban (129 males and 117 females) birds. However, as FID was not measured for all individuals, our dataset was reduced to 224 individuals, 85% of which were resighted during their first breeding. Although some individuals moved more than 10km, median dispersal distance was much shorter (322m), and most birds bred for the first time close to their natal areas (percentage of individuals remaining within 1 km of their natal sites: 75%; Fig 1A) and in the same habitat of birth (96% of dispersions were within the same habitat type). Movements between habitats were rare (10 individuals out of 224), and mainly from urban to rural areas (6 movements from urban to rural areas vs 3 movements from rural to urban ones).

### Correlates of natal dispersal

Sex, individual personality and habitat were the most important variables to explain interindividual differences in natal dispersal distances (Tables 1 and 2). Urban birds dispersed over shorter distances than rural ones, with females moving farther than males in both habitat types. Moreover, individuals with shorter FID dispersed over greater distances than their counterparts, regardless of their sex or the habitat where they were born (Fig 1B). Although social variables were weakly related to dispersal distances, individuals born in areas with low conspecific density tended to dispersed greater distances than those born in high-density areas (Table 2). Importantly, when habitat was removed from the analysis, conspecific density received stronger support as a predictor of dispersal distances, with individuals born in high-density areas dispersing less than those born in more isolated sites (Table 2). This change in the result suggests that habitat differences in dispersal distances are actually related to the higher conspecific density recorded in the surroundings of urban compared to rural sites (estimate: 13.16, 95% CI: 10.99–15.33). Finally, we found no differences in conspecific density or productivity between natal and dispersal areas in both habitat types (conspecific density: estimate: 2.44, 95% confidence interval: -5.58–1.35; conspecific productivity: estimate: 0.65, 95% confidence interval: -0.29–1.59). However, individuals dispersing farther settled in areas more similar in terms of conspecific densities than those staying close to their natal areas (estimate: -1.17, 95% CI: -2.20 - -0.15), a pattern not observed when considering changes in conspecific productivity (estimate: 0.27; 95% CI: -0.77–1.32). It is worth noting that similar results were obtained when restricting our dataset to individuals resighted during their first breeding attempt (S1 Table).

**Table 1:** Models obtained to assess the relative importance of individual’s traits (sex and personality, measured as FID), and social variables (conspecific density and productivity in the natal area) on the natal dispersal distances of rural and urban (habitat) burrowing owls *Athene cunicularia*. Models shown are the first 10 models ranked using their AICc. K: number of estimated parameters in approximating model, AICc: Akaike information criteria with small sample bias adjustment, ΔAICc: difference between each model and the best model (i.e., the model with the lowest AICc), weight: Akaike weight.

Model	K	AICc	ΔAICc	weight
FID + habitat + sex	6	590.51	0.00	0.25
conspeficic density + FID + habitat + sex	7	590.86	0.35	0.21
conspecific productivity + FID + sex + habitat	7	592.53	2.03	0.09
conspeficic density + FID + sex	6	592.93	2.42	0.07
conspecific productivity*habitat + FID*habitat + sex*habitat	10	593.07	2.56	0.07
conspeficic density + sex	5	593.16	2.65	0.07
habitat + sex	5	593.70	3.19	0.05
Sex	4	594.34	3.83	0.04
conspeficic density + habitat + sex	6	594.61	4.10	0.03
FID*habitat + sex*habitat	8	594.70	4.20	0.03

**Table 2:** Relative importance of individual’s traits (sex and personality, measured as FID), and social variables (conspecific density and productivity in the natal area) on the natal dispersal distances of rural and urban (habitat) burrowing owls *Athene cunicularia*. Estimates and 95% confidence intervals (2.5% and 97.5%) obtained after averaging models in Table 1 (all models) and using the subset of models that did not include habitat (models without habitat). We considered that a given variable has no, weak or strong support when the 95% confidence interval strongly overlapped with zero, barely overlapped with zero (asterisk), or did not overlap with zero (in bold), respectively. Results remained unchanged when model averaging was performed using all candidate models, not only those with ΔAICc < 2 (S3 Table).

**All models**			
**Variable**	**Estimate**	**2.50%**	**97.50%**
FID	-0.18	-0.34	-0.03
habitat (urban)	-0.49	-0.88	-0.09
sex (female)	0.63	0.40	0.87
conspecific density	-0.10	-0.25	0.05
**Models without habitat**			
**Variable**	**Estimate**	**2.50%**	**97.50%**
FID	-0.10	-0.23	0.03*
sex (female)	0.59	0.35	0.83
conspecific density	-0.14	-0.28	0.00

### Correlates of natal dispersal distances on productivity and survival

Birds breeding for their first time in rural areas were less productive than those inhabiting urban ones (Tables 3 and 4). However, when they dispersed farther from their natal areas, they raised more chicks during their first breeding attempt than those staying nearby, a relationship that was absent among urban individuals. Besides, females dispersing at larger distances of their natal areas also increased their productivity in the first breeding event. When considering the long term productivity of individuals (data available for 144 individuals), we found that all birds, urban and rural, tended to increase productivity with age and when at greater natal dispersal distances (Table 4). Results remained consistent when we repeated models using only individuals resighted during their first breeding attempt (S2 Table).

**Table 3:** Models obtained to assess the relationship between natal dispersal distances and productivity during the first breeding attempt, and long term productivity of rural and urban (habitat) burrowing owls *Athene cunicularia*. All models were run including year as a random term; models for long term productivity also included individual as a random term. Models shown are the first 10 models ranked using their AICc. K: number of estimated parameters in approximating model, AICc: Akaike information criteria with small sample bias adjustment, ΔAICc: difference between each model and the best model (i.e., the model with the lowest AICc), weight: Akaike weight.

**Productivity during the first breeding attempt**				
**Model**	**K**	**AICc**	**ΔAICc**	**weight**
sex + dispersal distance*habitat	7	1372.93	0.00	0.42
dispersal distance*sex + habitat	7	1374.79	1.86	0.17
dispersal distance*sex	6	1374.90	1.96	0.16
sex	4	1376.48	3.55	0.07
sex + habitat	5	1376.54	3.61	0.07
dispersal distance*habitat	6	1377.89	4.96	0.04
sex + dispersal distance + habitat	6	1377.99	5.06	0.03
sex + dispersal distance	5	1378.24	5.31	0.03
dispersal distance + habitat	5	1382.84	9.91	0.00
dispersal distance	4	1383.60	10.67	0.00
**Long term productivity**				
**Model**	**k**	**AICc**	**ΔAICc**	**weight**
sex + age	5	1239.58	0.00	0.39
sex + age + habitat	6	1240.69	1.11	0.22
sex + age + distst	6	1241.34	1.76	0.16
sex + age + distst + habitat	7	1242.15	2.57	0.11
sex + age + distst*habitat	8	1242.48	2.90	0.09
sex	4	1246.63	7.05	0.01
sex + habitat	5	1247.52	7.94	0.01
sex + distst	5	1248.16	8.58	0.01
sex + distst*habitat	7	1248.52	8.94	0.00
sex + distst + habitat	6	1248.64	9.06	0.00

**Table 4:** Estimates and 95% confidence intervals (2.5% and 97.5%) obtained after model averaging to assess the relationship between natal dispersal distances and productivity during the first breeding attempt, and long term productivity of rural and urban (habitat) burrowing owls *Athene cunicularia*. We considered that a given variable has no, weak or strong support when the 95% confidence interval strongly overlapped with zero, barely overlapped with zero (asterisk), or did not overlap with zero (in bold), respectively. Results remained unchanged when model averaging was performed using all candidate models, not only those with ΔAICc < 2 (S3 Table).

**Productivity during the first breeding attempt**			
**Variables**	**Estimate**	**2.50%**	**97.50%**
dispersal distance	-0.27	-0.82	0.28
sex (females)	0.50	0.13	0.87
habitat (rural)	-0.36	-0.76	0.04
dispersal distance*sex (females)	0.62	0.05	1.19
dispersal distance*habitat (rural)	0.42	0.11	0.72
**Long term productivity**			
**Variables**	**Estimate**	**2.50%**	**97.50%**
sex (female)	0.27	0.10	0.45
age	0.08	0.03	0.14
habitat (urban)	0.11	-0.11	0.34
dispersal distance	0.02	-0.06	0.11

**Fig 2:**
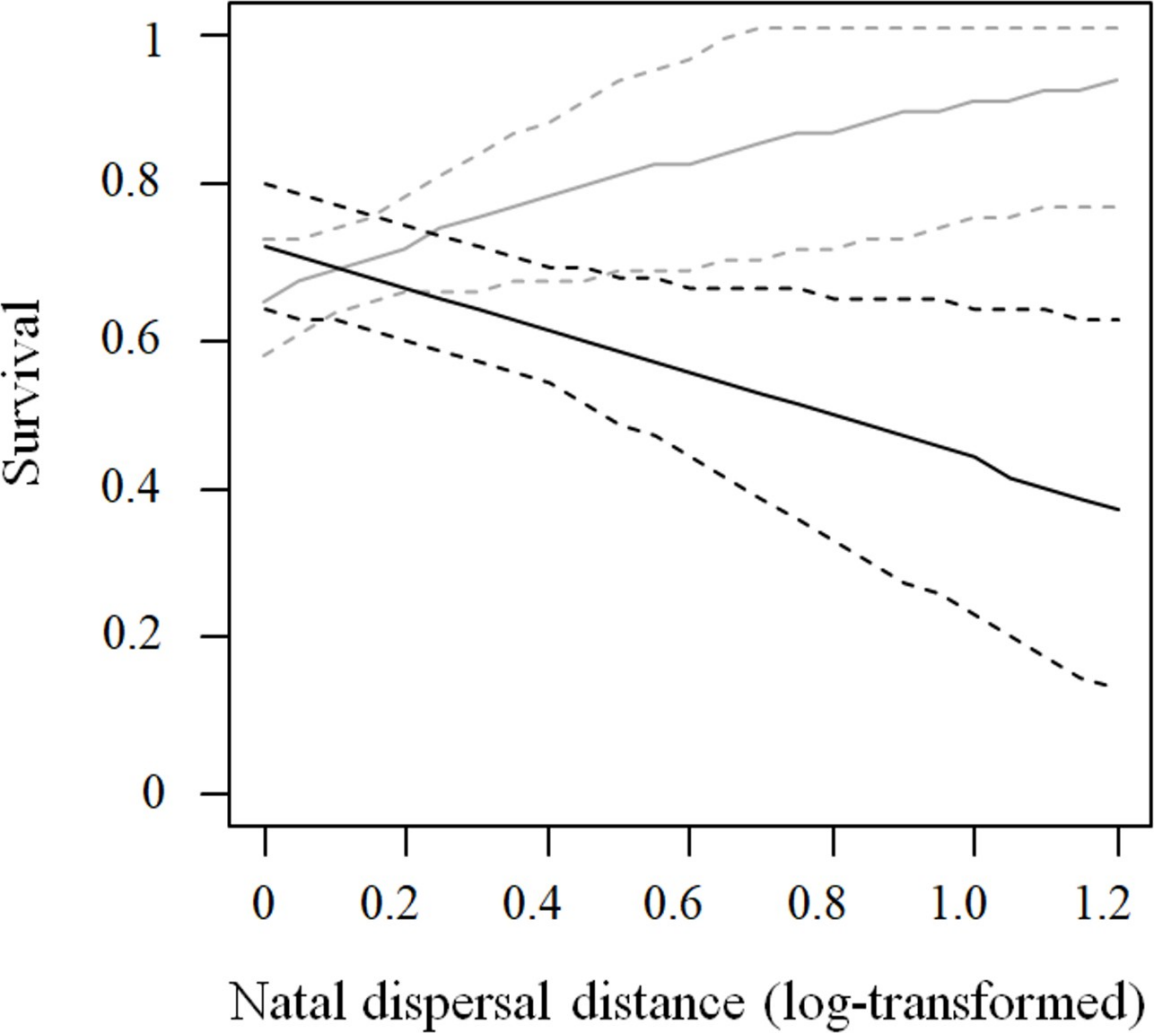
Relationship between natal dispersal and survival probabilities of male (gray lines) and female (black lines) burrowing owls *Athene cunicularia*. Solid lines represent the general tendency obtained using the first model shown in Table 5; dashed lines: 95% confidence intervals.

We estimated the effect of dispersal distances on future apparent survival probabilities using encounter histories of 248 individuals (1411 resightings). The overall goodness-of-fit test of the model was not statistically significant (χ^2^ = 34.34, d.f. = 43, p = 0.824). Model selection showed that resighting probabilities were lower for females (estimate: 0.82, 95% CI: 0.72–0.90) than for males (estimate: 0.93, 95% CI: 0.86–0.96; Table 5). Using this resighting structure, we tested the effect of natal dispersal distances on survival probabilities. The best model supported an interaction between dispersal distance and sex (Table 5), with females dispersing farther reducing their survival prospects compared to females staying closer (beta estimate: -1.21, 95% CI: -2.29 - -0.14). For males, future survival was not correlated with dispersal distances, as the estimate of the slope of this variable was not different to 0 (beta estimate: 1.60, 95% CI: -0.30–3.50; Fig 2). Habitat was not retained in models as a significant predictor of survival.

**Table 5:** Model comparison to assess the effects of natal dispersal distances (distance) on immediate survival probabilities of urban and rural (habitat) burrowing owls *Athene cunicularia*. K: number of estimated parameters in approximating model, AICc: Akaike information criteria with small sample bias adjustment, ΔAICc: difference between each model and the best model (i.e., the model with the lowest AICc), deviance: deviance explained by each model.

Survival model	Detection Probability	K	deviance	AICc	ΔAICc
distance*sex	sex	6	735.58	747.73	0.00
constant	sex	3	744.20	750.25	2.51
distance	sex	4	743.24	751.24	3.51
sex	sex	4	743.72	751.80	4.06
habitat	sex	4	744.17	752.25	4.51
distance*habitat	sex	6	740.19	752.35	4.62
distance+sex	sex	5	743.03	753.14	5.41
distance+habitat	sex	5	743.18	753.29	5.55
habitat+sex	sex	5	743.66	753.77	6.03
time	sex	12	729.65	754.24	6.50
time+sex	sex	13	728.62	755.30	7.57
habitat*sex	sex	6	743.57	755.73	7.99
ttime+habitat	sex	13	729.65	756.33	8.60
time+habitat+sex	sex	14	728.59	757.38	9.65
time*sex	sex	20	722.68	764.29	16.55
time*habitat	sex	21	722.75	766.51	18.78
time*habitat*sex	sex	36	703.86	781.10	33.37
time*habitat*sex	constant	35	707.06	782.01	34.28
time*habitat*sex	habitat+sex	37	703.84	783.39	35.65
time*habitat*sex	habitat	36	707.05	784.29	36.56
time*habitat*sex	habitat*sex	38	703.56	785.42	37.68
time*habitat*sex	time+sex	41	697.32	786.17	38.43
time*habitat*sex	time	40	700.19	786.70	38.96
time*habitat*sex	time+habitat+sex	42	697.31	788.51	40.78
time*habitat*sex	time+habitat	41	700.17	789.02	41.29
time*habitat*sex	time*sex	46	695.13	795.81	48.08
time*habitat*sex	time*habitat	46	697.52	798.21	50.47
time*habitat*sex	time*habitat*sex	57	682.28	809.86	62.13

## Discussion

Our results show that variability in the natal dispersal distances observed among burrowing owls was mainly explained by the additive effects of sex, personality and habitat. As expected, females moved farther distances than males in both habitat types, while urban birds (both males and females) dispersed over shorter distances compared to rural ones, partly because of the higher conspecific density recorded in urban compared to rural areas. Moreover, bold individuals—those with shorter FID—dispersed larger distances than their counterparts, regardless of their sex or the habitat and social environment in which they were born.

Sexual differences in natal dispersal distances are common among vertebrates to prevent mating between close relatives (inbreeding avoidance [66, 67, 68, 69]). However, the sex that disperses farther is not always the same and largely depends on the prevailing mating system of the species. In resource-defense mating systems, which is the prevailing system among birds, natal dispersal distances are generally shorter for males because they have to acquire and defend territories and, therefore, they may benefit from remaining near their natal area, where they are most familiar with resources and are probably best able to compete for them [49,70,71]. In our study species, males, but rarely females, actively defend an area larger than that immediately surrounding the nest burrow from conspecifics, with a more active response toward intruders at distances shorter than 100m [72]. This behavioral difference between sexes may underlie the sexual differences observed in the dispersal distances of individuals.

Sex is not the only individual trait affecting dispersal distances among burrowing owls. Previous findings showed that bolder, more asocial, and more active individuals can move greater distances and are more suited to colonizing new areas than shyer, social and less active ones [15,73,74]. In agreement with these results, we found a significant negative relationship between FID and natal dispersal among both urban and rural males and females. After settling in a breeding site, however, these bold individuals are more faithful than shy ones [39]. This apparent contradiction can be solved when considering the different behaviors correlated with FID. On the one hand, FID is positively correlated with explorative behavior [34]. Thus, bold individuals, which are also more explorative, can search for breeding sites exploring wider areas during their natal displacements than shy owls. When settled, however, bold individuals tend to remain in their breeding sites during consecutive years, even after suffering predation events that may cause their breeding failure [39]. A frequent finding from a wide range of vertebrate species is that individuals may change breeding sites after a poor breeding performance [75, 76, 77], or under predation pressures [78, 79, 80]. However, bolder individuals, which are also more aggressive toward predators [34], can choose to remain in their breeding site and cope with this important conditioning to take advantage of site familiarity.

Social variables received limited support in our modeling approach when we considered the habitat where individuals were born. However, after removing habitat from models, conspecific density became a strong predictor of dispersal distances, with individuals born in areas with low conspecific density covering greater distances than those born in high density areas. Thus, differences in natal dispersal patterns among urban and rural birds could be partially attributed to differences in conspecific density between habitats. Although high population densities can reduce individual fitness via increased competition for resources or direct interferences between individuals, thus promoting dispersal [81], our results did not support this hypothesis. Conversely, burrowing owls dispersed shorter distances when born in high-density areas. Several studies have shown that individuals use information about conspecifics (i.e., their presence, density or breeding performance) to predict habitat quality and thus select whether or not to settle [52, 82, 83, 84]. Young burrowing owls recruit into their breeding territories during their first year of life, so they are not able to use conspecific productivity as a proxy of habitat quality. Conversely, they can use conspecific density. Conspecific density can correlate with habitat quality due to the movement of individuals to high-quality patches and/or to the differential mortality of resident conspecifics. In our study species, predation is the main determinant of breeding failure [39, 41], so areas with a high density of conspecifics can represent areas where predation risk is rather low.

In vertebrates, current evidence suggests that natal dispersal could be penalized in terms of fitness [4], although some researches have reported opposite patterns [85, 86]. The low natal dispersal distances observed in our study suggest that moving far from areas where individuals were born is not the preferred strategy for burrowing owls. However, when analyzing the relationships between individual survival and breeding prospects, we found that females and rural individuals dispersing farther from their natal areas raised more chicks during their first breeding attempt than those staying close, a relationship that was absent among urban individuals. Moreover, long term productivity tended to increase when both urban and rural individuals dispersed at greater distances from their natal areas. Contrarily, females dispersing farther reduced their survival prospects compared to females staying closer and males. Thus, the reproductive benefits associated with large natal dispersal in females are outweighed by its survival costs [87, 88, 89]. This, along with the benefits obtained by males that stay close to natal areas, explain the low dispersal distances observed in the whole population. Although we cannot discard the possibility that the lower survival of females dispersing longer distances arises as a consequence of permanent emigration, the large size our study area (5400km^2^) and the intensive monitoring we performed (as shown by the large resignting probability observed for all individuals, independently of their sex and habitat) reduce this likelihood [4].

In conclusion, we found evidence supporting a role for individual traits (sex and personality) and conspecific density in explaining variability in the natal dispersal patterns of burrowing owls. Although urban areas per se did not affect this demographic parameter, the lower predation risk experienced by urban individuals may favor greater conspecific densities, which actually reduce dispersal propensity. From an evolutionary perspective, and although it is known that very low rates of dispersal among subpopulations are sufficient for the system to behave as a panmictic population [90], rates of dispersal among subpopulations determine the level of gene flow and could, therefore, affect processes such as local adaptation. Thus, the low natal dispersal distances combined with reduced breeding dispersal may be the underlying cause explaining the genetic structure detected among urban and rural populations of burrowing owls at small spatial scales [91]. Further research is needed to assess the generalizability of these results to other taxa to properly ascertain the consequences of urbanization in the ecology and evolution of species thriving in anthropogenic areas.

## Supporting information

S1 Table Relative importance of individual’s traits (sex and personality, measured as FID), and social variables (conspecific density and productivity in the natal area) on the natal dispersal distances of rural and urban (habitat) burrowing owls *Athene cunicularia*. These models were run using individuals resighted during their first breeding attempts (n = 189 individuals). Estimates and 95% confidence intervals (2.5% and 97.5%) were assessed after model averaging. We considered that a given variable has no, weak or strong support when the 95% confidence interval strongly overlapped zero, barely overlapped zero (asterisk), or did not overlap zero (in bold), respectively. Models shown are the first 10 models ranked using their AICc. Variable (*): model averaging performed using the subset of models that did not include habitat.
(DOCX)

S2 Table Relationship between natal dispersal distances and productivity during the first breeding attempt, and long term productivity of rural and urban (habitat) burrowing owls *Athene cunicularia*. These models were run using individuals resighted during their first breeding attempts (n = 189 individuals). Estimates and 95% confidence intervals (2.5% and 97.5%) were assessed after model averaging. We considered that a given variable has no, weak or strong support when the 95% confidence interval strongly overlapped zero, barely overlapped zero (asterisk), or did not overlap zero (in bold), respectively. All models were run including year as a random term; models for long term productivity also included individual as a random term. Models shown are the first 10 models ranked using their AICc.
(DOCX)

S3 Table Relative importance of individual’s traits (sex and personality, measured as FID), and social variables (conspecific density and productivity in the natal area) on the natal dispersal distances of rural and urban (habitat) burrowing owls Athene cunicularia. Estimates and 95% confidence intervals (2.5% and 97.5%) obtained after averaging all candidate models.
(DOCX)

S4 Table Estimates and 95% confidence intervals (2.5% and 97.5%) obtained after model averaging to assess the relationship between natal dispersal distances and productivity during the first breeding attempt, and long term productivity of rural and urban (habitat) burrowing owls Athene cunicularia. Estimates and confidence intervals were obtained after averaging all candidate models.
(DOCX)
