# Correction: Sex, personality and conspecific density influence natal dispersal with lifetime fitness consequences in urban and rural burrowing owls

**DOI:** 10.1371/journal.pone.0294485

**Published:** 2023-11-13

**Authors:** Álvaro Luna, Antonio Palma, Ana Sanz-Aguilar, José L. Tella, Martina Carrete

There are errors in the Funding section. The correct Funding statement is: The project was funded by Projects CGL2015‐71378‐P MINECO/FEDER, UE and CGL2012‐31888 and Fundación Repsol (José L. Tella and Martina Carrete). Antonio Palma and Álvaro Luna were supported by La Caixa‐Severo Ochoa International PhD Program (2014 and 2015 respectively). The funders had no role in study design, data collection and analysis, decision to publish, or preparation of the manuscript.
