# Supplementary material for: Sex, personality and conspecific density influence natal dispersal with lifetime fitness consequences in urban and rural burrowing owls
Source: PLoS One. 2020 Feb 12;15(2):e0226089. doi: 10.1371/journal.pone.0226089 (PMC7015421; doi:10.1371/journal.pone.0226089)
Supplement: S1 Table — These models were run using individuals resighted during their first breeding attempts (n = 189 individuals). Estimates and 95% confidence intervals (2.5% and 97.5%) were assessed after model averaging. We considered that a given variable has no, weak or strong support when the 95% confidence interval strongly overlapped zero, barely overlapped zero (asterisk), or did not overlap zero (in bold), respectively. Models shown are the first 10 models ranked using their AICc. Variable (*): model averaging performed using the subset of models that did not include habitat. (DOCX) [file pone.0226089.s001.docx]

**Table S1.** Relative importance of individual’s traits (sex and personality, measured as FID), and social variables (conspecific density and productivity in the natal area) on the natal dispersal distances of rural and urban (habitat) burrowing owls *Athene cunicularia*. These models were run using individuals resighted during their first breeding attempts (n=189 individuals). Estimates and 95% confidence intervals (2.5% and 97.5%) were assessed after model averaging. We considered that a given variable has no, weak or strong support when the 95% confidence interval strongly overlapped zero, barely overlapped zero (asterisk), or did not overlap zero (in bold), respectively. Models shown are the first 10 models ranked using their AICc. Variable (*): model averaging performed using the subset of models that did not include habitat.

| **Model** | **K** | **AICc** | **delta** | **weight** | **Variable** | **Estimate** | **2.50%** | **97.50%** |
| --- | --- | --- | --- | --- | --- | --- | --- | --- |
| FID + habitat + sex | 6 | 500.79 | 0.00 | 0.28 | FID | -0.18 | -0.34 | -0.02 |
| conspecific productivity + FID + sex + habitat | 7 | 502.45 | 1.66 | 0.12 | habitat (urban) | -0.54 | -0.94 | -0.14 |
| conspecific density + FID + sex | 6 | 505.89 | 1.87 | 0.14 | sex (females) | 0.68 | 0.42 | 0.94 |
| conspecific density + FID + habitat + sex | 7 | 502.78 | 1.98 | 0.10 | conspecific productivity | 0.06 | -0.08 | 0.19 |
| habitat + sex | 5 | 503.31 | 2.51 | 0.08 | conspecific density | -0.03 | -0.19 | 0.12 |
| conspecific productivity*habitat + FID*habitat + sex*habitat | 10 | 503.65 | 2.85 | 0.07 |  |  |  |  |
| sex (*) | 4 | 504.02 | 3.23 | 0.06 | **Variable** (*) | **Estimate** | **2.50%** | **97.50%** |
| habitat*sex | 6 | 504.06 | 3.27 | 0.05 | sex (females) | 0.61 | 0.35 | 0.88 |
| FID*habitat + sex*habitat | 8 | 504.33 | 3.54 | 0.05 | conspecific density | -0.09 | -0.24 | 0.06 |
| conspecific density + sex (*) | 5 | 504.99 | 4.20 | 0.03 | FID | -0.06 | -0.2’ | 0.08 |
| conspecific productivity + habitat + sex | 6 | 505.37 | 4.58 | 0.03 |  |  |  |  |
| conspecific density + habitat + sex | 6 | 505.39 | 4.59 | 0.03 |  |  |  |  |
| FID + sex (*) | 5 | 505.74 | 4.95 | 0.02 |  |  |  |  |
