# Supplementary material for: Sex, personality and conspecific density influence natal dispersal with lifetime fitness consequences in urban and rural burrowing owls
Source: PLoS One. 2020 Feb 12;15(2):e0226089. doi: 10.1371/journal.pone.0226089 (PMC7015421; doi:10.1371/journal.pone.0226089)
Supplement: S2 Table — These models were run using individuals resighted during their first breeding attempts (n = 189 individuals). Estimates and 95% confidence intervals (2.5% and 97.5%) were assessed after model averaging. We considered that a given variable has no, weak or strong support when the 95% confidence interval strongly overlapped zero, barely overlapped zero (asterisk), or did not overlap zero (in bold), respectively. All models were run including year as a random term; models for long term productivity also included individual as a random term. Models shown are the first 10 models ranked using their AICc. (DOCX) [file pone.0226089.s002.docx]

**Table S2.** Relationship between natal dispersal distances and productivity during the first breeding attempt, and long term productivity of rural and urban (habitat) burrowing owls *Athene cunicularia*. These models were run using individuals resighted during their first breeding attempts (n=189 individuals). Estimates and 95% confidence intervals (2.5% and 97.5%) were assessed after model averaging. We considered that a given variable has no, weak or strong support when the 95% confidence interval strongly overlapped zero, barely overlapped zero (asterisk), or did not overlap zero (in bold), respectively. All models were run including year as a random term; models for long term productivity also included individual as a random term. Models shown are the first 10 models ranked using their AICc.

| **Productivity during the first breeding attempt** | | | | | | | | |
| --- | --- | --- | --- | --- | --- | --- | --- | --- |
| **Model** | **k** | **AICc** | **∆AICc** | **weight** | **Variables** | **Estimate** | **2.50%** | **97.50%** |
| dispersal distance*sex + habitat | 6 | 661.19 | 0.00 | 0.43 | dispersal distance | -0.67 | -1.32 | -0.01 |
| dispersal distance*sex | 5 | 662.73 | 1.53 | 0.20 | sex (females) | 0.49 | 0.17 | 0.80 |
| sex + habitat | 4 | 663.84 | 2.64 | 0.11 | habitat (urban) | 0.29 | -0.02 | 0.61 |
| sex + dispersal distance*habitat | 6 | 663.87 | 2.68 | 0.11 | dispersal distance*sex (females) | 0.68 | 0.02 | 1.35 |
| sex + dispersal distance + habitat | 5 | 665.66 | 4.46 | 0.05 |  |  |  |  |
| sex | 3 | 665.71 | 4.52 | 0.05 |  |  |  |  |
| sex + dispersal distance | 4 | 667.42 | 6.22 | 0.02 |  |  |  |  |
| habitat | 3 | 668.04 | 6.85 | 0.01 |  |  |  |  |
| dispersal distance*habitat | 5 | 668.12 | 6.93 | 0.01 |  |  |  |  |
| dispersal distance + habitat | 4 | 670.12 | 8.93 | 0.01 |  |  |  |  |
| **Long term productivity** | | | | | | | | |
| **Model** | **k** | **AICc** | **∆AICc** | **weight** | **Variables** | **Estimate** | **2.50%** | **97.50%** |
| sex + age | 5 | 1239.58 | 0.00 | 0.39 | sex (female) | 0.27 | 0.10 | 0.45 |
| sex + age + habitat | 6 | 1240.69 | 1.11 | 0.22 | age | 0.08 | 0.03 | 0.14 |
| sex + age + dispersal distance | 6 | 1241.34 | 1.76 | 0.16 | habitat (urban) | 0.11 | -0.11 | 0.34 |
| sex + age + dispersal distance + habitat | 7 | 1242.15 | 2.57 | 0.11 | dispersal distance | 0.02 | -0.06 | 0.11 |
| sex + age + dispersal distance *habitat | 8 | 1242.48 | 2.90 | 0.09 |  |  |  |  |
| sex | 4 | 1246.63 | 7.05 | 0.01 |  |  |  |  |
| sex + habitat | 5 | 1247.52 | 7.94 | 0.01 |  |  |  |  |
| sex + dispersal distance | 5 | 1248.16 | 8.58 | 0.01 |  |  |  |  |
| sex + dispersal distance *habitat | 7 | 1248.52 | 8.94 | 0.00 |  |  |  |  |
| sex + dispersal distance + habitat | 6 | 1248.64 | 9.06 | 0.00 |  |  |  |  |
| dispersal distance *habitat | 6 | 1251.61 | 12.03 | 0.00 |  |  |  |  |
| dispersal distance + habitat | 5 | 1251.77 | 12.19 | 0.00 |  |  |  |  |
| dispersal distance | 4 | 1251.80 | 12.22 | 0.00 |  |  |  |  |
| null | 3 | 1252.59 | 13.01 | 0.00 |  |  |  |  |
| habitat | 4 | 1253.42 | 13.84 | 0.00 |  |  |  |  |
