# Supplementary material for: Sex, personality and conspecific density influence natal dispersal with lifetime fitness consequences in urban and rural burrowing owls
Source: PLoS One. 2020 Feb 12;15(2):e0226089. doi: 10.1371/journal.pone.0226089 (PMC7015421; doi:10.1371/journal.pone.0226089)
Supplement: S3 Table — Estimates and 95% confidence intervals (2.5% and 97.5%) obtained after averaging all candidate models. (DOCX) [file pone.0226089.s003.docx]

**Table S3. Relative importance of individual’s traits (sex and personality, measured as FID), and social variables (conspecific density and productivity in the natal area) on the natal dispersal distances of rural and urban (habitat) burrowing owls *Athene cunicularia*. Estimates and 95% confidence intervals (2.5% and 97.5%) obtained after averaging all candidate models.**

| **Variable** | **Estimate** | **2.50%** | **97.50%** |
| --- | --- | --- | --- |
| FID | -0.18 | -0.35 | -0.01 |
| habitat (urban) | -0.45 | -0.89 | -0.01 |
| sex (female) | 0.63 | 0.31 | 0.95 |
| conspecific density | -0.11 | -0.27 | 0.05 |
| conspecific productivity | 0.14 | -0.22 | 0.51 |
| habitat (urban)*conspecific productivity | -0.39 | -0.71 | -0.06 |
| habitat (urban)*FID | -0.01 | -0.34 | 0.33 |
| habitat (urban)*sex (female) | -0.09 | -0.72 | 0.55 |
| habitat (urban)*conspecific density | -0.13 | -0.45 | 0.20 |
