# Supplementary material for: Sex, personality and conspecific density influence natal dispersal with lifetime fitness consequences in urban and rural burrowing owls
Source: PLoS One. 2020 Feb 12;15(2):e0226089. doi: 10.1371/journal.pone.0226089 (PMC7015421; doi:10.1371/journal.pone.0226089)
Supplement: S4 Table — Estimates and confidence intervals were obtained after averaging all candidate models. (DOCX) [file pone.0226089.s004.docx]

**Table S4. Estimates and 95% confidence intervals (2.5% and 97.5%) obtained after model averaging to assess the relationship between natal dispersal distances and productivity during the first breeding attempt, and long term productivity of rural and urban (habitat) burrowing owls *Athene cunicularia*. Estimates and confidence intervals were obtained after averaging all candidate models.**

| **Productivity during the first breeding attempt** | | | |
| --- | --- | --- | --- |
| **Variable** | **Estimate** | **2.50%** | **97.50%** |
| dispersal distance | -0.24 | -0.79 | 0.32 |
| sex (females) | 0.49 | 0.13 | 0.85 |
| habitat (rural) | -0.35 | -0.75 | 0.05 |
| dispersal distance*sex (females) | 0.62 | 0.05 | 1.19 |
| dispersal distance*habitat (rural) | 0.42 | 0.11 | 0.72 |
| **Long term productivity** | | | |
| **Variables** | **Estimate** | **2.50%** | **97.50%** |
| sex (female) | 0.27 | 0.09 | 0.45 |
| age | 0.08 | 0.03 | 0.14 |
| habitat (urban) | 0.13 | -0.10 | 0.37 |
| dispersal distance | 0.05 | -0.07 | 0.17 |
| dispersal distance*habitat (urban) | -0.12 | -0.28 | 0.05 |
